# Supplementary material for: Visualization of Murine Vascular Remodeling and Blood Flow Dynamics by Ultra-High-Frequency Ultrasound Imaging
Source: Int J Mol Sci. 2022 Oct 31;23(21):13298. doi: 10.3390/ijms232113298 (PMC9655559; doi:10.3390/ijms232113298)
Supplement: Supplementary file 1 [file ijms-23-13298-s001.zip › ijms-1927438-supplementary.pdf]

Index Supplemental Appendix

**Title**	**Page number**
Supplementary Figure S1	2
Supplementary Figure S2	3, 4
Supplementary Figure S3	5
Supplementary Figure S4	6
Supplementary Figure S5	7
Supplementary Figure S6	8
Supplementary Figure S7	9

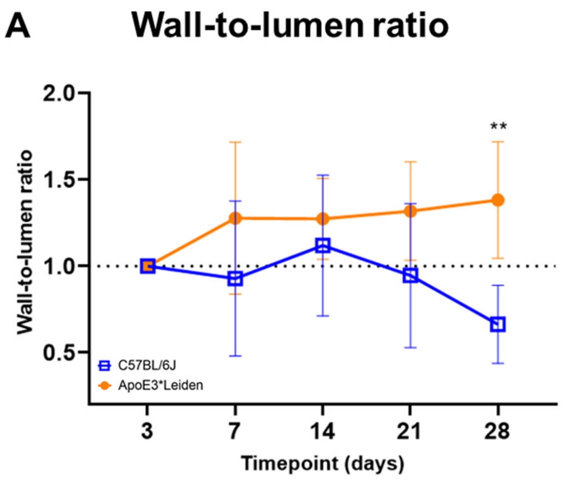

Supplementary Figure S1 | Wall-to-lumen ratio of vein grafts in C57BL/6J and ApoE3*Leiden mice over time.

(**A**) Wall-to-lumen ratio of C57BL/6J (n=7) and ApoE3*Leiden (n=7) mice over the course of 28 days post-surgery. The data are shown relative to baseline t=3 days after surgery, with lumen and wall size being 100% (ratio=1.0). Statistics: ANOVA mixed model, corrected for multiple testing; ** $p<0.01$.

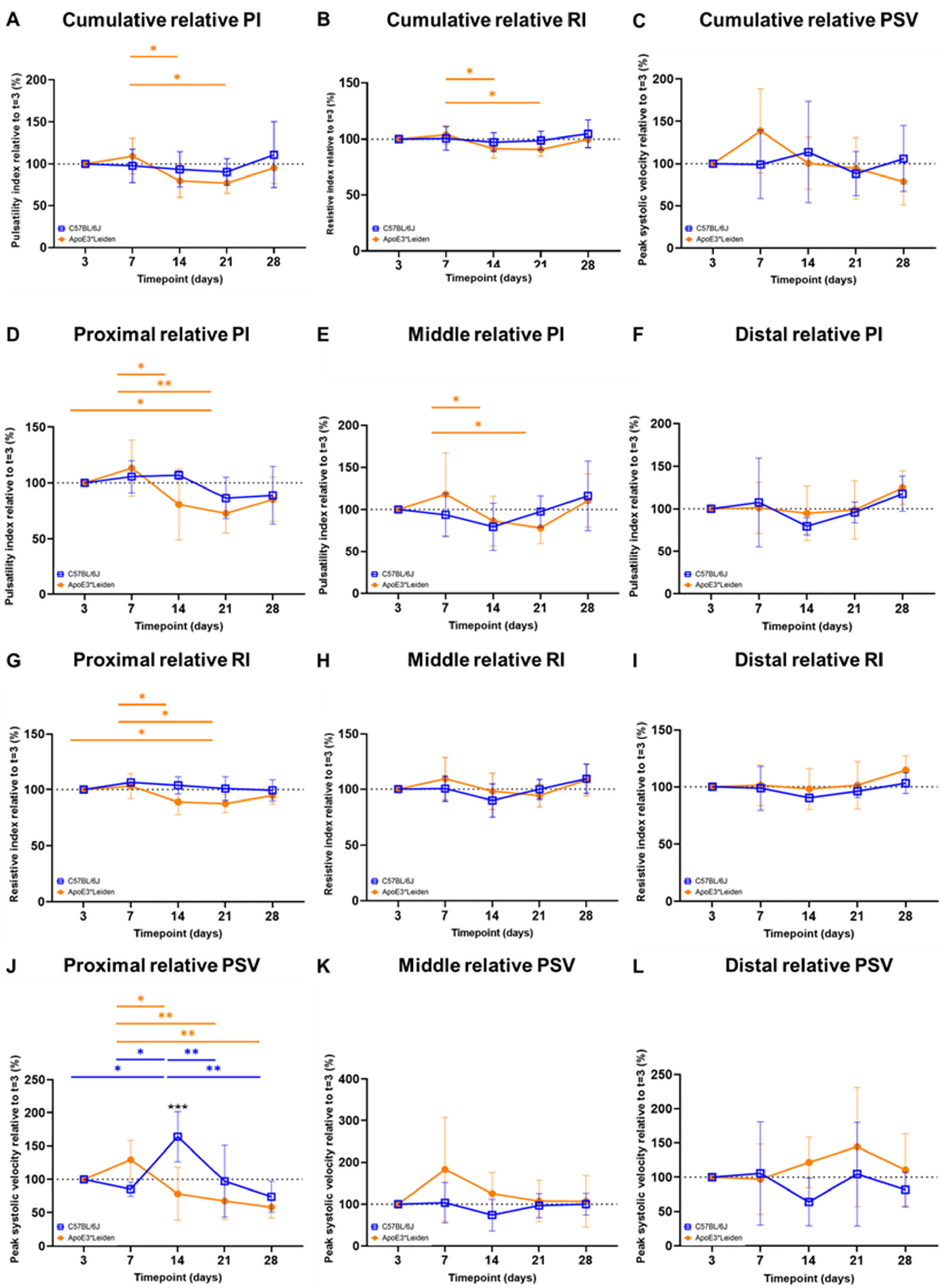
A
Cumulative relative PI
Pulsatility index relative to t=3 (%)
B
Cumulative relative RI
Resistive index relative to t=3 (%)
C
Cumulative relative PSV
Peak systolic velocity relative to t=3 (%)
D
Proximal relative PI
E
Middle relative PI
F
Distal relative PI
G
Proximal relative RI
H
Middle relative RI
I
Distal relative RI
J
Proximal relative PSV
K
Middle relative PSV
L
Distal relative PSV
Timepoint (days)
C57BL/6J
ApoE3*Leiden

Supplementary Figure S2 | Velocity measurements of vein grafts in C57BL/6J and ApoE3*Leiden mice over time, as split per proximal, middle, and distal location.

(**A, B, C**) Cumulative values for respectively the PI, RI, and peak systolic velocity (PSV) of vein grafted C57BL/6J (n=7, chow diet) and ApoE3*Leiden mice (n=7, high fat diet) over time. Pulsatility index (PI) (**D, E, F**) , resistive index (RI) (**G, H, I**), and peak systolic velocity (PSV) (**J, K, L**) of vein grafts in C57BL/6J and ApoE3*Leiden mice during 28 days after surgery. The data are shown relative to baseline t=3 days after surgery, with lumen and wall size being 100%. Statistics: ANOVA mixed model, corrected for multiple testing; * $p<0.05$, ** $p<0.01$.

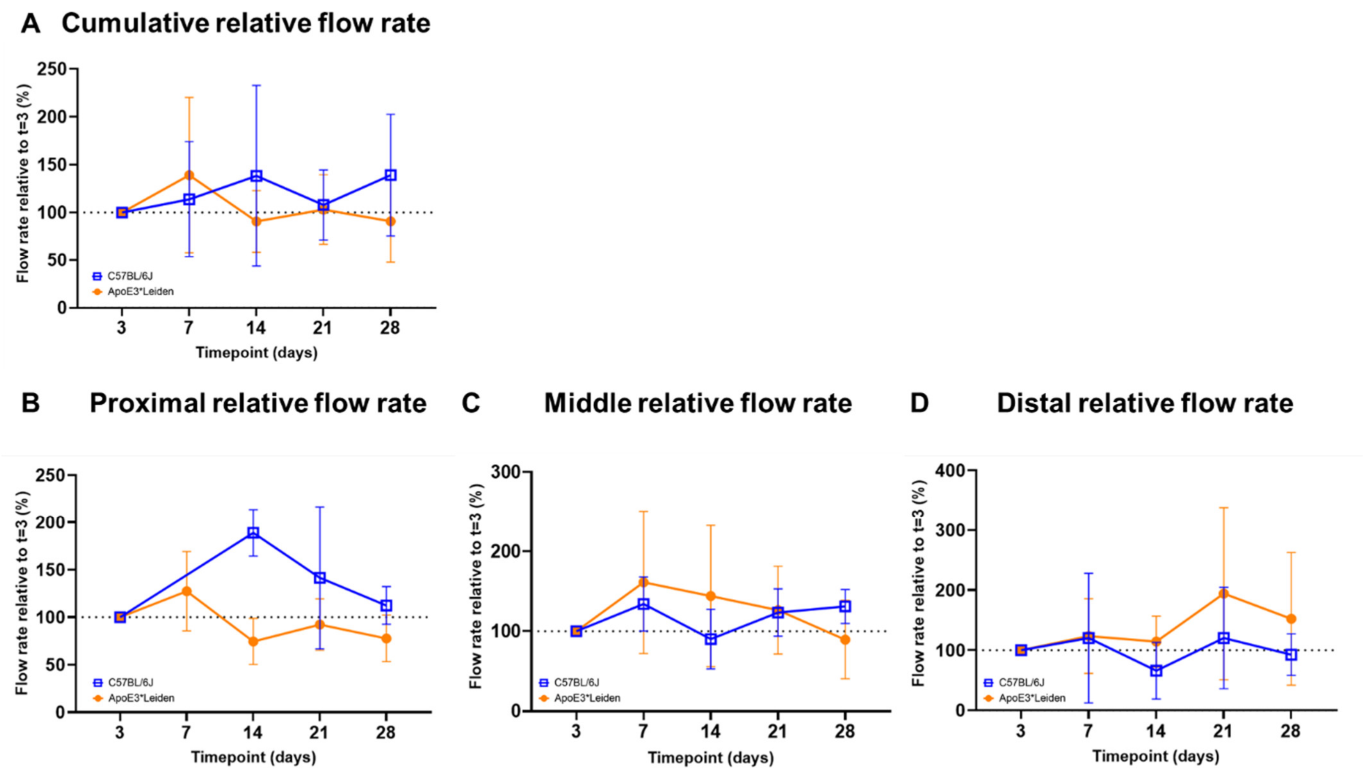

Supplementary Figure S3 | Relative blood flow rate of vein grafts in C57BL/6J and ApoE3*Leiden mice over time, as split per proximal, middle, and distal location.

Cumulative (**A**), proximal (**B**), middle (**C**), and distal (**D**) relative flow rate of vein grafts in C57BL/6J and ApoE3*Leiden mice during 28 days post-surgery. The data are shown relative to baseline t=3 days after surgery, with lumen and wall size being 100%. Statistics: ANOVA mixed model, corrected for multiple testing.

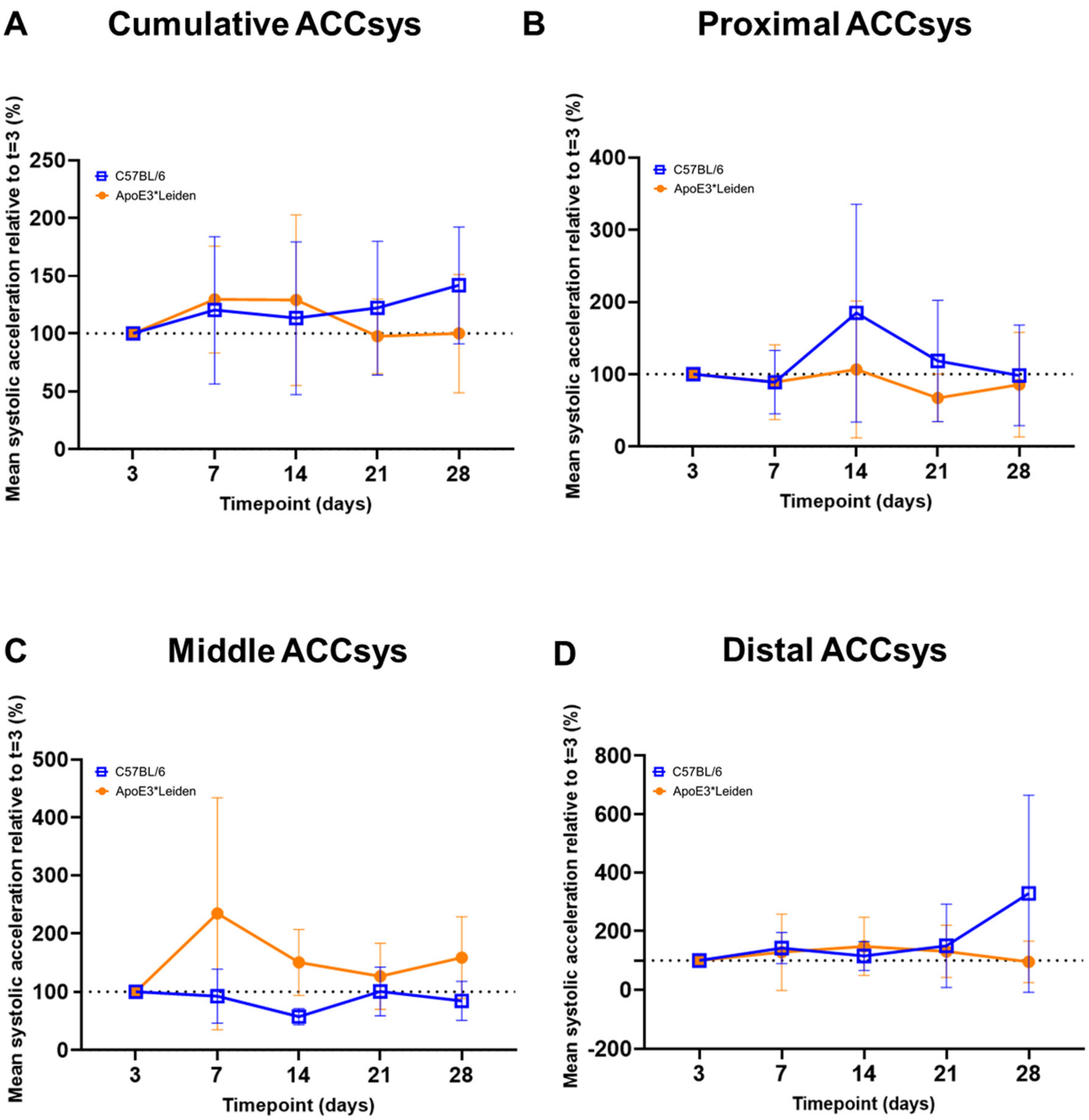

Supplementary Figure S4 | Mean systolic acceleration (ACCsys) in vein grafts of C57BL/6J and ApoE3*Leiden mice.

Cumulative (**A**), Proximal (**B**), Middle (**C**), and Distal (**D**) ACCsys in vein grafts of C57BL/6J and ApoE3*Leiden mice from 3 to 28 days post- surgery. Statistics: ANOVA mixed model, corrected for multiple testing.

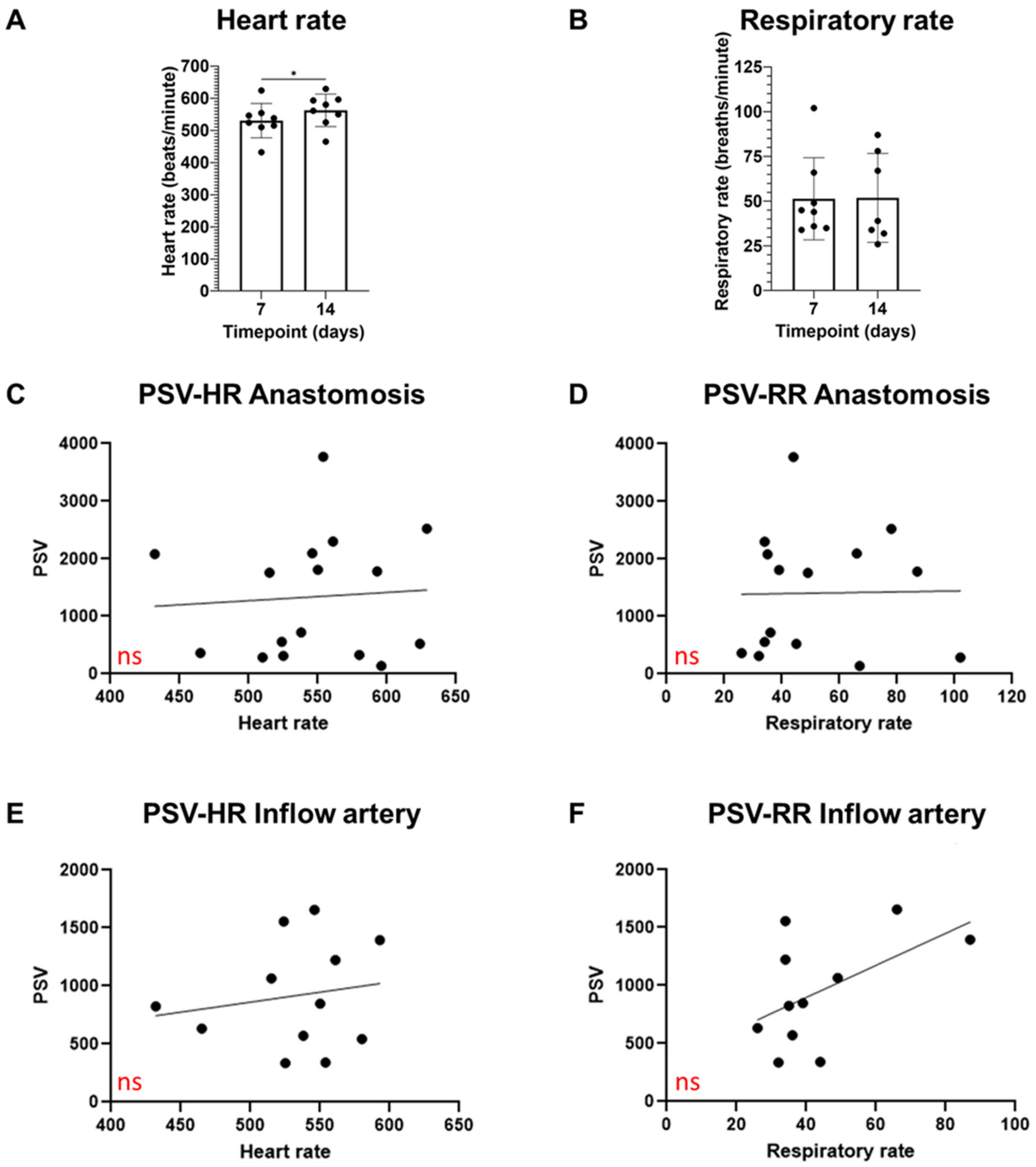

Supplementary Figure S5 | Heart and respiratory rate measures as related to the PSV in C57BL/6J mice that underwent AVF surgery.

Heart (**A**) and respiratory (**B**) rate of C57BL/6J mice (n=8) on time points 7 and 14 post AVF surgery. Correlation plots of the peak systolic velocity (PSV) at the anastomotic site of the AVF against the heart (**C**) and respiratory (**D**) rate (time point 7 and 14 days combined). Correlation plots of the PSV at the anastomotic site of the AVF against the heart (**E**) and respiratory (**F**) rate (time point 7 and 14 days combined). Statistics: Paired T-test and Pearson r; * $p<0.05$. ns=not significant.

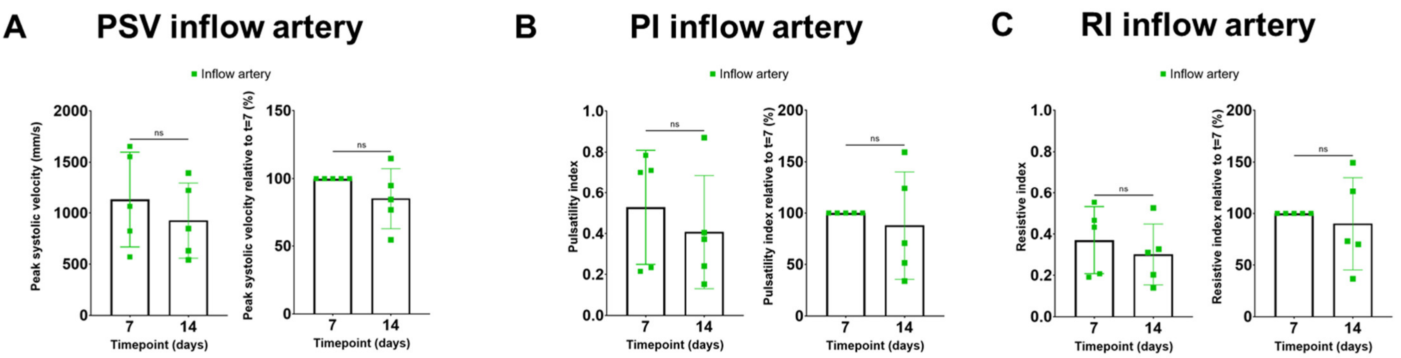

Supplementary Figure S6 | Absolute and relative velocities of the inflow artery of C57BL/6J AVF mice over time.

Absolute and relative peak systolic velocity (PSV) (**A**), pulsatility index (PI) (**B**), and resistive index (RI) (**C**) in the inflow artery of C57BL/6J AVF mice at 7 and 14 days post-surgery (n=5) in C57BL/6J mice after 7 and 14 days post-surgery. Statistics: Paired T-test. ns=not significant.

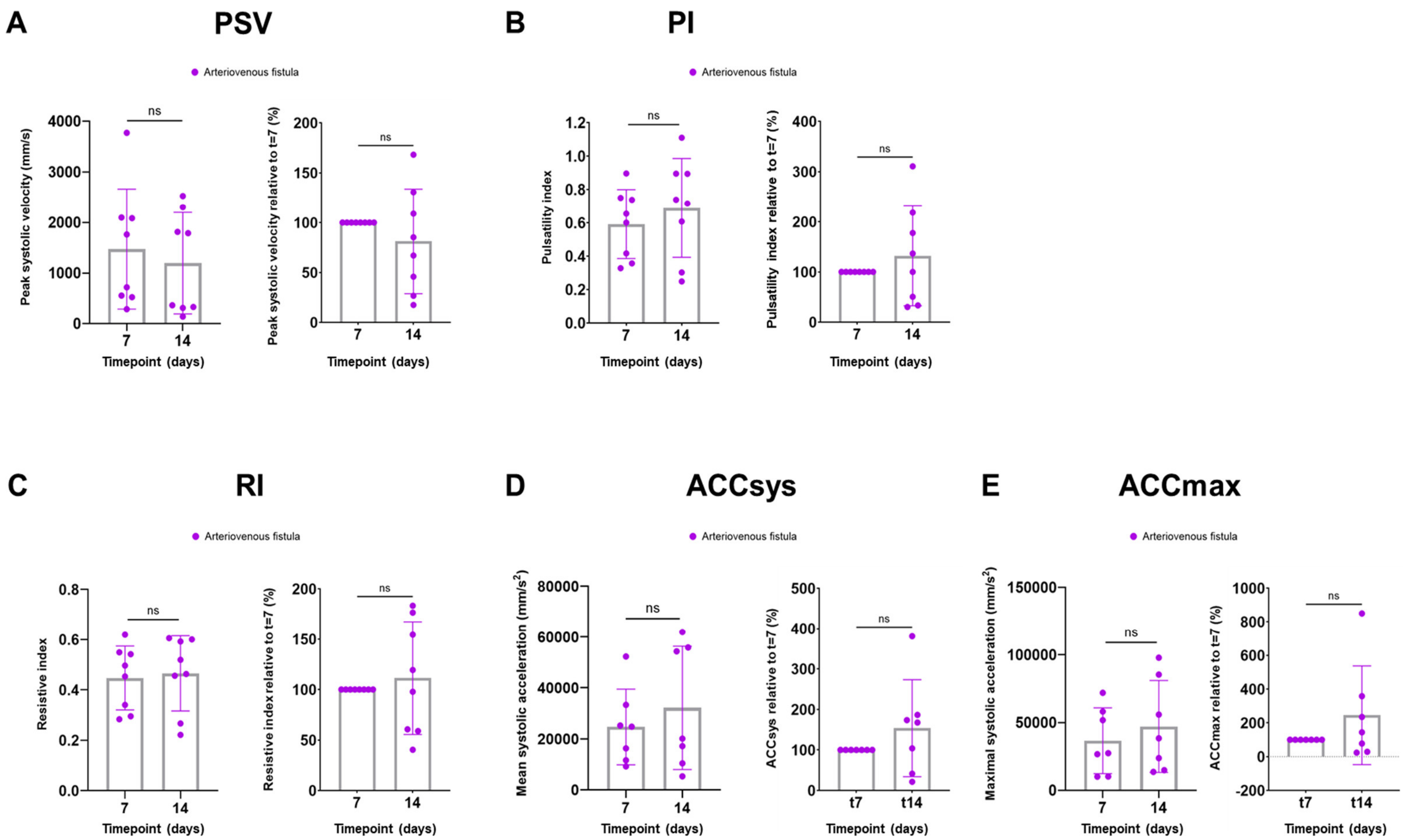

Supplementary Figure S7 | Velocity and acceleration measurements of arteriovenous fistulas in C57BL/6J mice over time.

Relative and absolute values of the peak systolic velocity (**A**), pulsatility index (**B**), resistive index (**C**), mean systolic acceleration (**D**), and maximal systolic acceleration (**E**) in AVFs in C57BL/6J mice after 7 and 14 days post-surgery (n=7). Paired T-test. ns=not significant.
